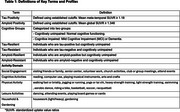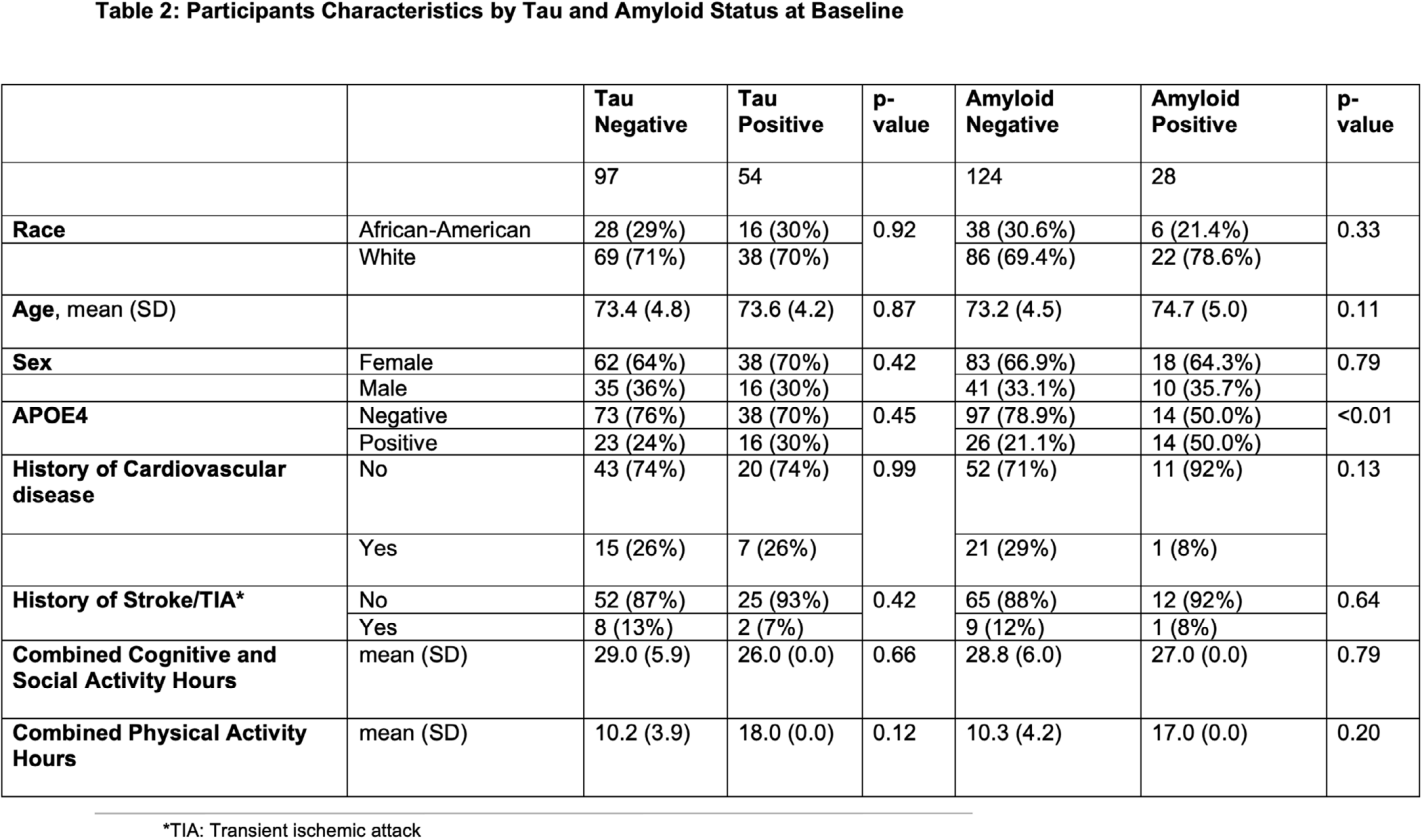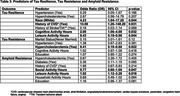# Social, Physical, and Cognitive Activity Patterns and Their Association with Tau and Amyloid Resistance and Resilience

**DOI:** 10.1002/alz70860_107660

**Published:** 2025-12-23

**Authors:** Daniel Willie‐Permor, Oscar L Lopez, Steven E. Reis, Anum Saeed, Beth E. Snitz, Brian J Lopresti, Victor L Villemagne, M. Ilyas Kamboh, Neelesh Nadkarni, C. Elizabeth Shaaban, Ann D Cohen

**Affiliations:** ^1^ Alzheimer's Disease Research Center (ADRC), PITTSBURGH, PA, USA; ^2^ University of Pittsburgh, Pittsburgh, PA, USA; ^3^ University of Pittsburgh Alzheimer's Disease Research Center (ADRC), Pittsburgh, PA, USA; ^4^ University of Pittsburgh School of Medicine, Pittsburgh, PA, USA; ^5^ University of Pittsburgh, School of Medicine, Pittsburgh, PA, USA; ^6^ Department of Human Genetics, University of Pittsburgh, Pittsburgh, PA, USA

## Abstract

**Background:**

Some individuals escape the development of Alzheimer's disease (AD) amyloid and tau pathology suggesting resistance, and not all individuals with these pathologies develop cognitive impairment (CI), suggesting resilience. Social, physical, and cognitive activities may impact resistance and resilience, and this study explores their roles.

**Method:**

This is a secondary analysis of existing data from the Heart Strategies Concentrating on Risk Evaluation (Heart SCORE) study. Multiple activities were collected via the CHAMPS Activities Questionnaire. We grouped them into five domains: social, cognitive, physical, leisure, and household/gardening Total weekly hours were calculated for each domain. We also created combined cognitive‐social activities and combined physical activities. Tau burden was measured using [18F]Flortaucipir (FTP) PET imaging, and amyloid using [11C] Pittsburgh Compound‐B (PiB) PET imaging (Table 1). Cognition was categorized clinically as either unimpaired cognition (CU) or impaired cognition (MCI/dementia). Resilience and resistance profiles were defined as follows: tau‐resilient: tau‐positive, CI‐negative; tau‐resistant: tau‐negative, CI‐negative and amyloid resistant). We used logistic regression to identify activity predictors of tau and amyloid resistance and resilience.

**Result:**

The study included 152 participants, with demographic characteristics stratified by tau and amyloid status at baseline (Table 2). Throughout the period of follow‐up, greater cognitive activity was significantly associated with tau resilience, while leisure activity was inversely associated with tau resilience (Table 3) Among demographic and health factors, throughout the follow‐up, being White and having a history of cardiovascular disease significantly predicted tau resilience. Hypercholesterolemia was significantly and inversely associated with tau resistance. Increased leisure activity hours were significantly associated with amyloid resistance, while social activity showed an inverse association. Among demographic and health predictors, none were significant, though diabetes was associated with reduced amyloid resistance while education was positively associated (Table 3). Amyloid resilience could not be included due to model non‐convergence secondary to low power.

**Conclusion:**

Cognitive activities and leisure activities may contribute to tau resilience and amyloid resistance respectively. Promoting a multifaceted approach to activity engagement and cardiovascular risk‐factor control may be key in mitigating the impact of tau and amyloid pathology on cognitive health.